# Metagenomic Investigation Uncovers Presence of Probiotic-Type Microbiome in Kalparasa^®^ (Fresh Unfermented Coconut Inflorescence Sap)

**DOI:** 10.3389/fmicb.2021.662783

**Published:** 2021-08-13

**Authors:** Murali Gopal, Sandip Shil, Alka Gupta, K. B. Hebbar, M. Arivalagan

**Affiliations:** ^1^ICAR-Central Plantation Crops Research Institute, Kasaragod, India; ^2^Research Centre, ICAR-Central Plantation Crops Research Institute, Mohitnagar, India

**Keywords:** coconut inflorescence sap, Kalparasa^®^, metagenome, West Coast Tall, probiotic-type microbiome

## Abstract

The phloem sap tapped from unopened inflorescence (spadix) of coconut palm using a novel collecting device, “coco-sap chiller,” has been branded Kalparasa^®^ (henceforth as *Kalparasa* in the text) to distinguish its properties not found in sap harvested by traditional methods. To know its hitherto unidentified microbiome profile, we employed high-throughput sequencing to uncover the bacteriome and mycobiome in fresh and 12-h fermented samples. Fresh *Kalparasa* had a pH of 7.2, which dropped to 4.5 after 12 h, signifying fermentation of the sap. Diversity analysis indicated fresh *Kalparasa* having higher bacterial species than the fermented one. Contrary to this, fresh sap had lower fungal/yeast diversity than the fermented sample. Fresh *Kalparasa* had relatively higher abundance of probiotic-type *Leuconostoc* genus followed by equal proportions of *Gluconobacter*, *Acetobacter*, and *Fructobacillus*. The 12-h fermented *Kalparasa* showed a significant increase in *Gluconobacter* with a sharp decrease in *Leuconostoc*. Mycobiome data revealed fresh *Kalparasa* to be preponderant in *Saccharomyces* and *Hanseniaspora* genera of yeasts while the fermented sap had higher representation of *Hanseniaspora* and *Cortinarius* and lesser *Saccharomyces*. This suggested that the fermentation of *Kalparasa* was probably driven by symbiotic culture of bacteria and yeasts (SCOBY), particularly acetic acid bacteria and non-*Saccharomyces* yeasts. The bacteriome-function predictions highlighted the enrichment of glycerophospholipid, ABC transporters, purine, and pyrimidine metabolisms. Based on our findings, *Kalparasa* containing large population of *Leuconostoc mesenteroides*, *Fructobacillus fructosus*, *Saccharomyces cerevisiae*, and *Hanseniaspora guilliermondii* can be promoted as a healthy “unfermented” plant edible food containing live probiotic-type microbiome during its consumption.

## Introduction

Xylem and phloem saps are watery fluids (Barnes, 1893) having a definitive role in biological functions of plant growth, signaling, and reproduction. Whereas xylem sap transports mainly water and dissolved minerals from roots to leaves and other parts of the plants (Sperry, 2003) and maintain the hydraulic connectivity of plants between the soil and the atmosphere (Steppe et al., 2015), phloem sap transports photosynthate (sugars) from and within the source tissues (leaves) and to the sink tissues (non-photosynthetic tissues) (Halford, 2010). Owing to their richness in sugars, amino acids, vitamins, and minerals, both xylem and phloem saps are exploited by humans as edible plant saps for fresh and fermented drinks and in processed form as syrups, sugars, and sweeteners (Svanberg et al., 2012). Among the phloem saps, those from palms such as palmyra palm (*Borassus flabellifer*) (Le et al., 2020), coconut palm (*Cocos nucifera* L.) (Hebbar et al., 2015), African oil palm (*Elaeis guineensis*) (Djeni et al., 2020), and date palm are being increasingly used for producing several value-added food products for human consumption.

The coconut inflorescence sap (vernacularly termed *neera*) is generally collected in open mud pots or plastic containers. The collection methods and collecting units such as open earthen pots traditionally used (Vidanapathirana et al., 1983) make it vulnerable to microbial and insect contamination resulting in fermentation and poor/unpalatable quality sap collection (Tauro and Rao, 1964; Vidanapathirana et al., 1983; Nakamura et al., 2004; Hebbar et al., 2015). It has been reported that date palm sap collected using open clay pots contaminated with Nipah virus (NiV) by secretions and excretions of bat *Pteropus* spp. was one of the main reasons for the spread of deadly NiV in Bangladesh (Rahman et al., 2011; Islam et al., 2016), highlighting the health hazards associated with traditional collection methods. Thus, it became imperative to know the quality of coconut inflorescence sap in order to assure an edible product.

Non-availability of proper collection apparatus and fermentation inhibitors resulting in quick conversion to alcoholic beverage of the coconut inflorescence sap made its tapping an offense in many of the coconut-growing states in southern India as per the Excise Acts (Hebbar et al., 2015). The palm tree was designated as “excise tree” (under Section 11A of The Central Excise Act, 1944) and was permitted to be tapped only in a limited numbers accompanied with proper license. This age-old prohibition became a handicap for coconut farmers to earn several times higher remuneration from coconut cultivation *via* tapping of fresh *neera* and converting it to value-added products such as sugars, gurs (traditional Indian sugar), and vinegar.

With the recent development of a simple and innovative *neera* collection device, coco-sap chiller (under Indian patent consideration) by ICAR-Central Plantation Crops Research Institute (Hebbar et al., 2015), the possibility of tapping fresh, hygienic, and unfermented *neera* by a larger community of coconut farmers has now become practical, which could help them earn many-fold higher compared to sales of mature coconuts. A conservative estimate claims 36,000 million Indian Rupees could be generated annually if just 10% of palms of 2 million ha in India are tapped at one litre Kalparasa/palm/day (Hebbar et al., 2015). Besides the large economic returns, the coco sap chiller also offers an enclosed collection system that minimizes the chance of contamination from bats, rodents, etc., reducing the possibility of any health hazards As a result, some southern states in India have amended the Excise law by removing coconut *neera* tapping from the prohibitory list, allowing coconut farmer producer organizations to harvest the sap, but with certain caveats. The *neera* collected using the coco-sap chiller is unique when compared with traditional methods of collection and, therefore, has been named Kalparasa^®^ (Trade Mark No. 2813919 under Trademarks Registry of Intellectual Property India), literally meaning “juice or flavor of the *Kalpavriksha*—the coconut palm.” Significant differences in the physical, chemical, biochemical, and microbiological properties of Kalparasa^®^ (henceforth as *Kalparasa* in text) and traditionally collected *neera* in earthen pot were recently reported (Hebbar et al., 2020).

An important area of research in edible plant saps is on their microbiota profile, which is known to play a critical role in determining sap quality characteristics (Tamang et al., 2016; Perry and Fiore, 2020). In-depth studies of maple sap microbiota had indicated that yeasts related to *Mrakia* sp., *Mrakiella* sp., and *Guehomyces pullulans* were dominant and stable fungal microbiota, while *Pseudomonas fluorescens* was the one from the bacterial community (Filteau et al., 2011). *Pulque* is an alcoholic drink made by fermentation of *aguamiel*, which is a fresh sap collected from many different agave plant species growing in Mexico. 16S rRNA cloning studies of *pulque* showed that the genera *Lactobacillus* and *Leuconostoc* of Firmicutes and *Zymomonas*, *Acetobacter*, and *Gluconobacter* of Proteobacteria were the most abundant in this fermented beverage (Escalante et al., 2004). A possibility of distinct lineage of *Zymomonas mobilis* was recently reported in *pulque* based on shotgun metagenomic studies of five stages of the fermented sap combined with whole-genome shotgun sequencing of the isolated bacteria (Chacón-Vargas et al., 2020). In a more updated deep sequencing study of the *pulque*, earlier unreported bacteria such as *Sphingomonas* and *Weissella* were also reported (Rocha-Arriaga et al., 2020). Recently, the indigenous microbial communities present in the bark, sap, and soil of cider gum *Eucalyptus gunnii* were analyzed through amplicon-based phylotyping to identify the bacteria and fungi associated with the natural fermentation of sap from this tree consumed as a mild alcoholic drink, *way-a-linah*, by the Aborigines of Australia (Varela et al., 2020). They reported the presence of the fungal genus *Lachancea* in abundance in the sap samples.

Because *neera* from coconut was prone to quick and natural fermentation, several studies were conducted to understand the microbial properties of the palm inflorescence sap. Rao et al. (1947) were one of the earlier researchers who reported the nutritional requirement for growing large-sized yeast isolated from coconut toddy. A more detailed report of the population of bacteria and yeast colonies to the tune of 10^6^ cells/ml in fresh coconut *neera* was later reported (Tauro and Rao, 1964). As coconut *neera* had wide adoption in Sri Lanka, microbial research focused on coconut sap fermentation particularly in relation to yeasts present in the coconut wine (Jayatissa et al., 1978). Later, the changes undergone in the sap from the time of collection to storage for 5 days detailing the microbial succession during the initial lactic acid fermentation followed by alcoholic fermentation and finally acetic fermentation were reported (Vidanapathirana et al., 1983; Atputharajah et al., 1986). More recently, the probiotic properties of the lactic acid bacteria (LAB) such as *Lactobacillus brevis* (Accession number: MH74860) isolated from the naturally fermenting coconut *neera* were reported (Somashekaraiah et al., 2019). Fermented coconut palm wine, tuba, consumed in Mexico was analyzed for the bacterial diversity using 16S rRNA amplicon sequencing and was reported to have *Fructobacillus* as the main genus in all samples followed by *Leuconostoc, Gluconacetobacter, Sphingomonas*, and *Vibrio* (Astudillo-Melgar et al., 2019). Nevertheless, all the above studies were performed with the coconut sap collected by traditional methods that had no or little control in keeping the freshness of the fluid while it was being collected.

However, with the development of the innovative coco-sap chiller for collection of coconut *neera* by ICAR-CPCRI (Hebbar et al., 2015), the health-promoting quality parameters of *Kalparasa* were found to be significantly superior in physical, biochemical, and microbiological properties than the traditional collection method (Hebbar et al., 2020). *Neera* collected in the coco-sap chiller had a higher pH and Brix percentage, almost nil alcohol, and lower bacterial and yeast populations compared to traditionally collected *neera* in earthen pots. Advanced chromatographic instrumentation (UPLC coupled with TQD-MS/MS) studies could highlight the superior biochemical properties of *Kalparasa* in terms of sugars, amino acids, vitamins, phenols, and flavonoids (Hebbar et al., 2020). Similarly, metabolome study of *Kalparasa* using LC-MS revealed its richness in secondary metabolites that had the potential to aid in green synthesis of nanoparticles (Rajesh et al., 2020). The fermentation kinetics in terms of Vit C and sugar degradation was recently published for *Kalparasa* analyzed at different time periods and stored under different conditions (Pandiselvam et al., 2021). With increasing adoption of the ICAR-CPCRI technology, both entrepreneurs and consumers are keen to know the biological properties including microbial contents of *Kalparasa*.

The microbial profile of *Kalparasa*, which is a critical determinant of its quality, thus far had been studied mainly using conventional culture-dependent techniques to assess the bacterial and fungal communities present in the fresh sap and while the changes are occurring during its natural fermentation (Hebbar et al., 2020; Pandiselvam et al., 2021). Although the conventional culture-dependent technique enables physical availability of microbiota, it is known to provide less than 1% information of the microbial diversity because of innate difficulties associated with isolation methods. However, with the advent of advanced culture-independent molecular methods, in-depth information of microbial diversity is now fathomable at the metagenome level.

We hypothesized that a metagenome analysis could reveal the full-scale microbial diversity of fresh unfermented *Kalparasa*, particularly the probiotic-type bacteria and fungi that could improve our knowledge of the important microbial property of this health drink. Toward proving our hypothesis, we undertook the culture-independent metagenomic approach of analyzing the bacterial and fungal microbiome with 16S rRNA and ITS amplicon sequencing, respectively, of fresh unfermented *Kalparasa* and report the outcome in this article, probably the first of such on coconut inflorescence sap.

## Materials and Methods

*Kalparasa* was harvested from three uniformly aged, healthy, yield-stabilized West Coast Tall variety coconut palms growing in the Institute farm. The palms were cultivated in red sandy loam soil. Integrated nutrient management with application of 500:320:1200 g NPK and 25 kg organic manure in two split doses was adopted. Phosphorus fertilizer application was skipped sometimes as per requirement, Tractor plowing of the interspaces after monsoon to clear the weeds and sprinkler irrigation during the non-monsoon period (October through May) was regularly done. The pH of the soil was 5.6 with 0.6% OC content. As the palms were not affected by any serious pests or diseases, no major plant protection measures were taken for at least 3 years prior to tapping of the palms other than preventive cares.

The collection of *Kalparasa* for metagenomic analysis was initiated during the November to December period as per the standardized protocol described using the coco-sap chiller (Hebbar et al., 2015). In addition, to minimize the possibility of entry of any microbial contaminants, the following precautions were taken: (i) surface sterilization by swabbing of 70% ethanol of the spadix, internal and outside surfaces of tubular arm of the coco-sap chiller, and the connection tube draining the sap into the polythene bag; (ii) autoclaved polythene bag for collection of the sap; and (iii) hand sanitization by the sap collectors during fixing of the coco-sap chiller and then removal of the polythene bag containing the *Kalparasa* from the coco-sap chiller.

### Study of Aerial Microbiota of the Palm Canopy

While the coco-sap chiller was being fixed to the spadix, a survey was carried out to capture the aerial microbiota present in the canopy environment of the palm. Different microbiological media agar plates (plate count agar for bacteria, Sabouraud’s Dextrose agar for fungi and yeasts, and Kenknight and Munaiers agar for actinomycetes) were strategically placed on suitable canopy parts (mainly fronds) for about 15 min to allow deposition of any aerially moving microbiota that could enter the coco-sap chiller while fixing it to spadix. At least two plates for each agar medium were placed. The plates were closed with lids after the exposure period, placed into sterile polythene bags, tied and sent down to ground, and were immediately transferred to the microbiology laboratory for the incubation at 28°C ± 2°C for 48–72 h for scoring the growth of bacterial and fungal colonies and 5 days for actinomycetes colonies.

### Microbial Analysis of *Kalparasa*

The polythene bag containing the *Kalparasa* harvested overnight was removed from the coco-sap chiller, brought down from the palm, and right away transferred to the microbiology laboratory. The pH and color of the samples were immediately checked to confirm the quality of the *Kalparasa*. A sensory evaluation was also done to assure that the sap was fresh, aromatic, and unfermented. About 50 ml of the sub-sample from each bag was simultaneously taken out for culture-dependent microbial analysis. About 50 ml (×2) of the samples was transferred to sterile centrifuge tubes (50 ml) and kept at –45°C for the culture-independent metagenome study. Another 250 ml of the sub-sample was kept in a sterile plastic bottle for overnight fermentation (12 h). Thus, we had two different treatments to analyze: fresh and 12-h fermented *Kalparasa*. The next morning, 50 ml of sub-sample from each of the three bottles was immediately subjected to culture-dependent microbial analysis and 50 ml was transferred to sterile centrifuge tubes and stored at –45°C for the metagenomic study.

For culture-dependent analysis, 10-ml aliquots of *Kalparasa* from freshly collected and 12-h fermented samples were serially diluted and pour plated in different agar media to enumerate the bacterial, fungal, yeast, and actinomycetes populations. The plates were incubated for different time periods and then observed for colony-forming units of the microbial communities.

For metagenomic analysis, 50 ml of fresh and 12-h fermented *Kalparasa* samples was used for total DNA extraction using the DNeasy Power Food Microbial kit (Qiagen) following the manufacturer’s protocol. The extracted DNA was quantified using Nanodrop (Thermo Scientific Inc, Wilmington, DE, United States) and Qubit (Invitrogen, Carlsbad, CA, United States) and further processed immediately for sequencing. Paired end (250 × 2) 16S rRNA amplicon sequencing of the V3–V4 region was performed for the bacterial diversity analysis of the *Kalparasa*. The primers employed for amplifying the V3–V4 variable region were 341F: CCTACGGGNGGCWGCAG and 805R: GACTACHVGGGTATCTAATCC; for the ITS: ITS1F: CTTGGTCATTTAGAGGAAGTAA and ITS2R: GCTGCGTTCTTCATCGATGC. To begin, for having sufficient concentrations of the target fragments, repeated amplification for each sample was done with the following PCR conditions: pre-amplification denaturation at 95°C for 3 min; 25 cycles (35 cycles for ITS) of denaturation at 95°C for 30 s, annealing at 55°C for 30 s, and extension at 72°C for 30 s; and extension at 72°C for 10 min followed by holding at 4°C. The amplicons were then pooled in equimolar concentrations, cleaned up, purified using Agencourt AM pure magnetic beads (Beckman Coulter, Brea, CA, United States), and then quantified using Qubit. The pooled PCR amplicon samples were sequenced on Illumina HiSeq following the sequencing procedure of Caporaso et al. (2012).

### Statistical Bioinformatic Analysis

#### Pre-processing and Statistical Analysis

The raw 16S rRNA and ITS amplicon sequencing data for bacteria and fungi, respectively, were processed using the DADA2 v1.10.1 analysis pipeline package for modeling and correcting Illumina-sequenced amplicon errors (Callahan et al., 2016) in R v 3.6.0 environment (R Core Team, 2019). The workflow of the DADA2 R package processed the reads in order of stages: filtering, dereplication, chimera identification, and merging paired-end reads. In order to filter the data for high quality (HQ), the de-multiplexed reads were sieved using the parameters maxN = 0, maxEE = 2, and TruncQ = 2 for quality threshold as suggested in Edgar and Flyvbjerg (2015). The cutoff values for quality trimming are -250 and 250 bp for bacterial forward and reverse reads, respectively, and -250 and 240 bp for fungal forward and reverse reads, respectively, so that these cutoffs removed the poor-quality sequences having a phred score of less than 20. Finally, de-multiplexed HQ data were inferred into an amplicon sequence variant (ASV) table, which is a higher-resolution analog of the traditional operational taxonomic units (OTUs) table. Taxonomies of the ASVs (for bacterial and fungal reads) were assigned based on picking up close reference protocol against the Greengenes database Consortium (Version 13.8) at 97% sequence identity cutoff (DeSantis et al., 2006) and the latest UNITE general FASTA release for Fungi (Sun et al., 2020), respectively.

The de-multiplexed HQ processed data were further handled using the Bioconductor package phyloseq v1.26.0 (McMurdie and Holmes, 2013). In order to filter out low-occurrence/poorly represented ASVs from the bacterial reads, the ASVs that do not appear more than 10 times in more than half the samples were further removed, since they were essentially noise variables. However, in the case of fungal reads, no such operation was performed. The practice of data normalization and rarefaction to the smallest sample size is statistically inefficient and, therefore, inadvisable (McMurdie and Holmes, 2014). Hence, the data were not further normalized for comparisons. Alpha diversity measures of richness (like Chao1, ACE, and Fisher) were estimated over the samples. In order to compute beta diversity, principal coordinate analyses (PCoAs) based on weighted-UniFrac distance were performed and plotted. UniFrac distance measure was preferred due to the fact that this measure takes care of the phylogeny relationships of microbiome. Also, significant differences among beta diversity indices were evaluated using a paired Wilcoxon test for the bacterial and fungal communities across the samples, CS-F and CS-12H. The relative abundances of individual taxa were analyzed for statistically significant differences between the CS-F and CS-12H samples (bacterial and fungal) using the Bioconductor package DESeq2 v1.24.0 (Love et al., 2014).

#### Functional Metagenomic Content Prediction

In order to predict the functional metagenomic content of the bacterial community over the samples, the ASVs were submitted to the Piphillin server (Narayan et al., 2020), which is based on the relative abundance of the 16S rRNA sequences corresponding to genomes in regularly updated, functionally annotated genome databases. Finally, feature and pathway tables containing predicted Kyoto Encyclopedia of Genes and Genomes (KEGG) ortholog (KO) occurrence were retrieved using a 99% cutoff threshold and the KOs were also annotated using the KEGG database (Kanehisa and Goto, 2000).

### Statistical Analysis

All statistical computations were performed using R packages (R Core Team, 2019). Bacterial and fungal diversity analysis was carried out by standard richness indices and principal component analysis. Bacterial metagenome functions were extracted using KEGG annotations and enrichment data.

## Results

A total of six *Kalparasa* samples, three fresh (CS-F1, CS-F2, and CS-F3) and three fermented for 12 h (CS-12H1, CS-12H2, and CS-12H3), were analyzed for microbial properties using culture-dependent and -independent methods. Before their analysis, the microbial profile of the aerial environment of the coconut canopy was determined using a culture-dependent procedure. The coconut canopy aerial microbiota on the day of fixing the coco-sap chiller was found to be sparse for all the three palms (West Coast Tall var.) selected for *Kalparasa* tapping. Exposure of agar plates to catch the airborne microbiota in the coconut canopy resulted in the growth of one bacterial, one fungal, and three yeast colonies in the respective media (data not furnished). On the other hand, microbial status of the freshly collected and 12-h fermented Kalparasa indicated the presence of bacteria (CS-F: 2×10^5^, CS-12H: 4×10^6^ cfu ml^–1^) and yeasts in both samples. The population of yeasts was significantly higher in the fermented samples (CS-12H: 15×10^7^ cfu ml^–1^) compared to fresh Kalparasa (CS-F: 6×10^4^ cfu ml^–1^). The pH of the fresh Kalparasa was 7.2, and it dropped to 4.5 after 12 h of keeping in ambient conditions.

### Bacteriome of *Kalparasa*

**FIGURE 1:**
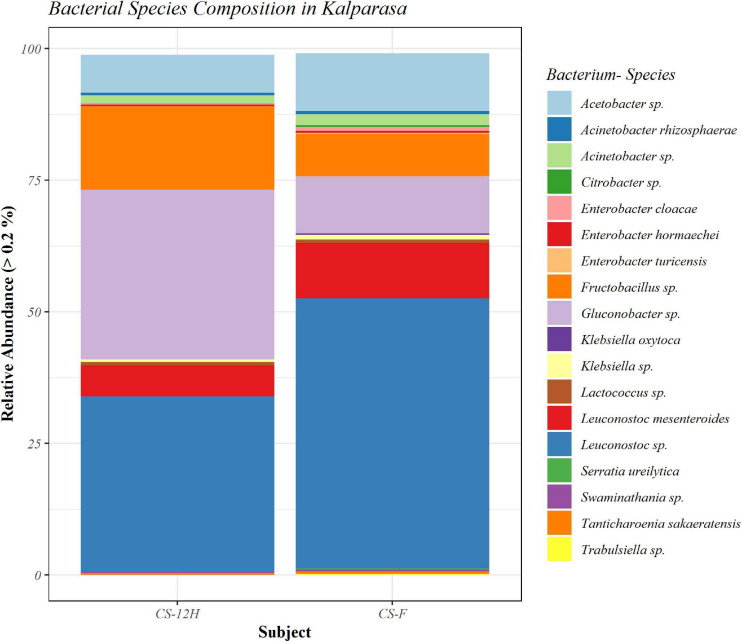
Bar plot representing relative abundance percentage (>0.2%) of detected bacterial species in fresh (CS-F) and fermented (CS-12H) *Kalparasa*.

**FIGURE 2:**
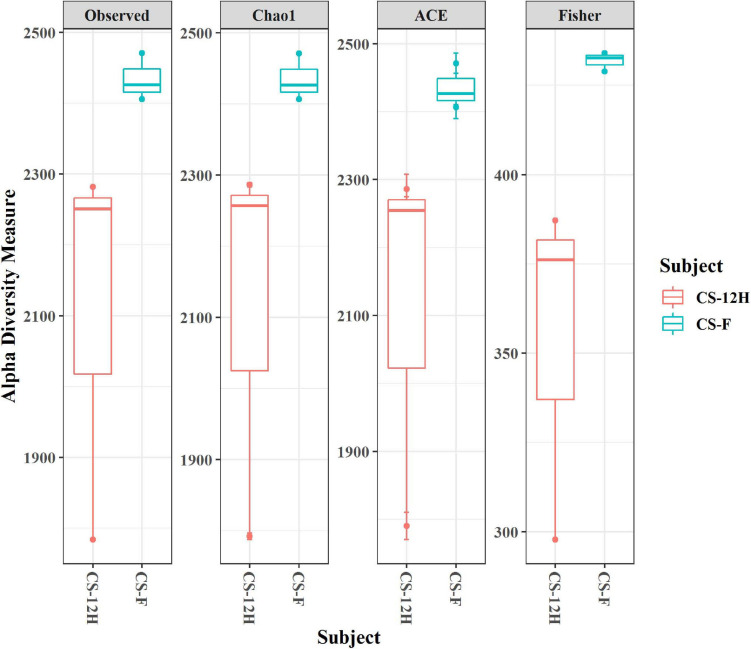
Alpha diversity indices (for observed, Chao1, ACE, and Fisher measures) for bacterial reads in fresh (CS-F) and fermented (CS-12H) *Kalparasa*.

**FIGURE 3:**
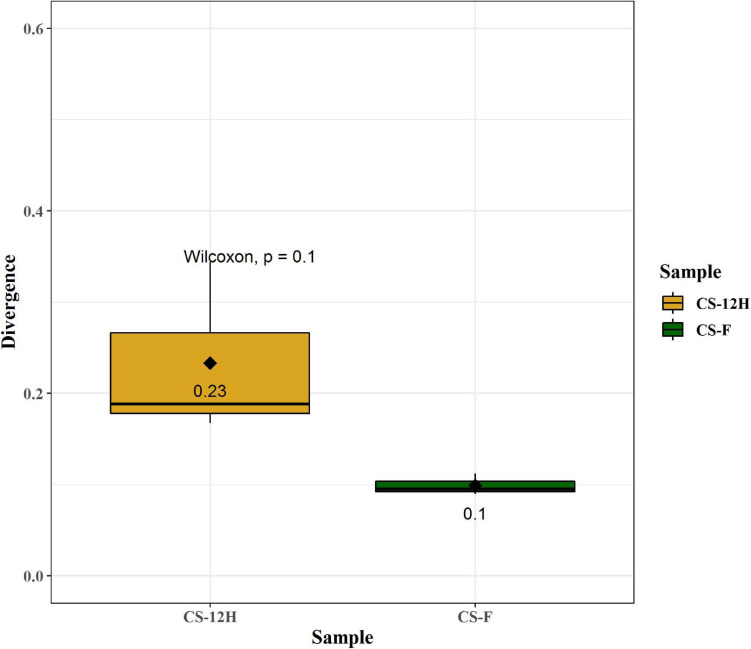
Quantification of bacterial divergence (heterogeneity/spread) within given samples by the mean sample dissimilarity or beta diversity. Taking average, over all pairwise dissimilarities is sensitive to sample size and heavily biased as the similarity values are not independent. To reduce this bias, the dissimilarity of each sample against the group mean is calculated. Here, CS-F sample divergence is 0.1, whereas CS-12H sample divergence is 0.23. These can be compared between groups in order to compare differences in group homogeneity.

**FIGURE 4:**
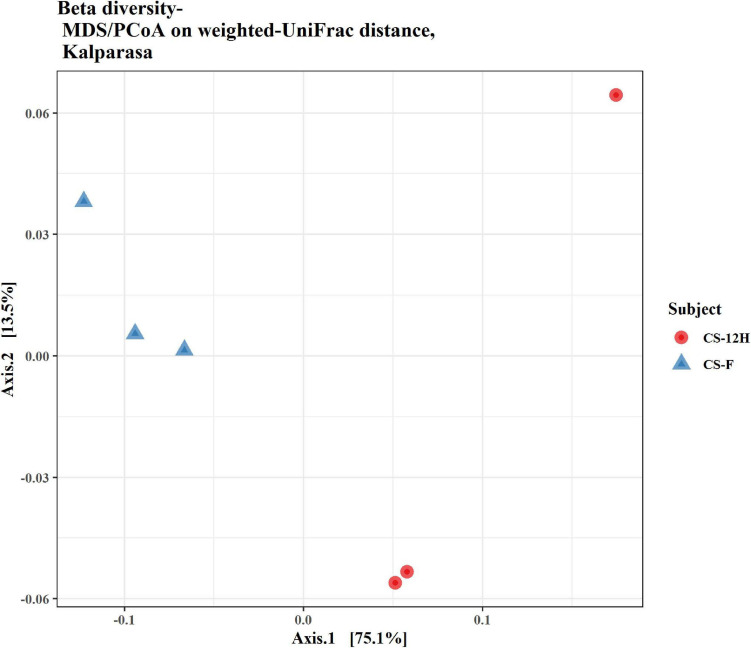
Beta diversity/dissimilarity unifrac distance measure and principal coordinate analysis (PCoA) for bacterial reads in fresh (CS-F) and fermented (CS-12H) *Kalparasa*.

For culture-independent metagenomic profiling of *Kalparasa*, the V3–V4 variable region of 16S rRNA was amplified for all the six samples. After pre-processing the raw data, a total of 1,192,109 chimera-free reads were obtained (Table 1). A Venn diagram presents the distribution of all identified bacterial ASVs of CS-F, CS-12H, and their interactions. The CS-F had 71 unique ASVs whereas CS-12H had only 44 unique ASVs (Supplementary Figure 1). Only two bacterial phyla, Firmicutes (70.4% in CS-F, 55.65% in CF-12H) and Proteobacteria (28.6% in CS-F and 43.06% in CS-12H), were found to be present in the *Kalparasa* samples while Actinobacteria and Bacteroidetes were not detected. We could identify a total of three classes, four orders, five families, 16 unique bacterial genera, and 18 species in the samples (Supplementary Table 1). The fresh unfermented *Kalparasa* (CS-F) had highest abundance of the genus *Leuconostoc* (61.15%) followed by *Acetobacter* (10.8%), *Gluconobacter* (10.49%), and *Fructobacillus* (7.74%). The 12-h fermented *Kalparasa* (CS-12H) had a much lower *Leuconostoc* content (39.3%) and lower *Acetobacter* (7.20%) but three times more *Gluconobacter* (31.52) and twice higher *Fructobacillus* (15.47%) compared to fresh *Kalparasa* (Supplementary Table 2). Within the *Leuconostoc* genus, *Leuconostoc mesenteroides* was conspicuous and it also followed the same pattern with a decrease in its relative abundance from CS-F to CS-12H (Figure 1). The relative abundance and Log2-fold changes of phyla in fresh and fermented *Kalparasa* showed that latter samples had 8.08 and 7.93 times more Firmicutes and Proteobacteria than the fresh sample (Supplementary Figure 2), while the changes in genera are furnished in Supplementary Figure 3. The diversity indices of the bacterial richness and divergence shown in terms of alpha (Figure 2) and beta (Figure 3) diversity clearly indicated the higher richness of bacterial genera in fresh *Kalparasa* assessed for Observed, Chao, ACE, and Fisher measures (Supplementary Table 3). The beta diversity showed that CS-F sample divergence was 0.1, whereas CS-12H sample divergence was 0.23. The PCoA with unifrac distance indicated that wide differences existed between the taxonomic compositions of the CS-F and CS-12H sample set, as the first coordinate discriminated them perfectly. The *x*-axis informed that this separation explained about 75.1% of the variance in the whole dataset (Figure 4).

**TABLE 1:** Bacterial preprocessing reads count reduction information for *Kalparasa* samples using the DADA2 R package.

Sample ids	No. of input reads (raw)	No. of reads after filtering	No. of reads after merging	No. of reads after removal of chimeras
*CS-12H1	469,464	395,521	353,833	188,840
CS-12H2	537,018	447,676	406,929	218,607
CS-12H3	499,694	421,198	380,531	197,721
**CS-F1	399,548	337,152	290,634	179,176
CS-F2	445,641	353,676	305,171	194,033
CS-F3	466,546	390,462	342,894	213,732

*CS-12H (*Kalparasa* sample kept in ambient conditions for 12 h). **CS-F (fresh *Kalparasa*).

### Mycobiome of *Kalparasa*

**FIGURE 5:**
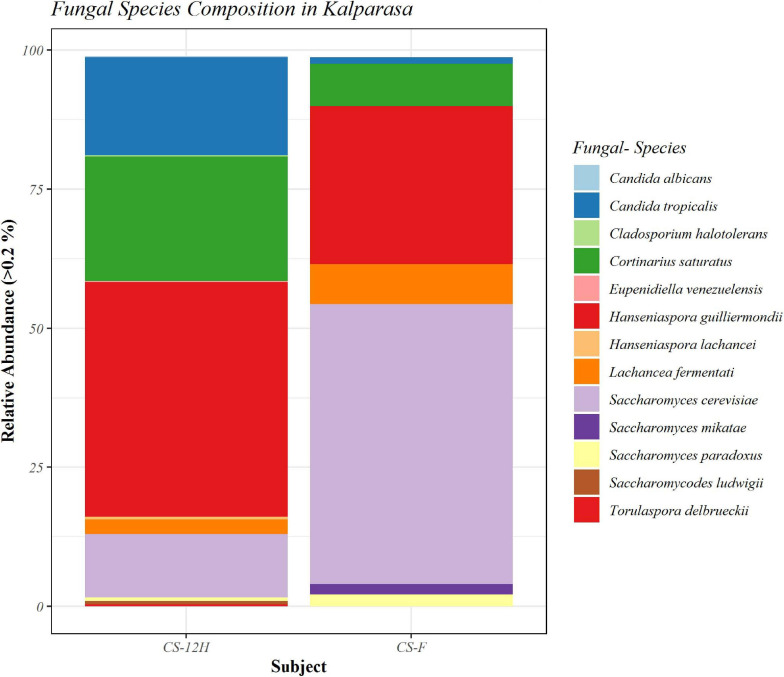
Bar plot representing relative abundance percentage (>0.2%) of detected fungal species in fresh (CS-F) and 12-h fermented (CS-12H) *Kalparasa*.

**FIGURE 6:**
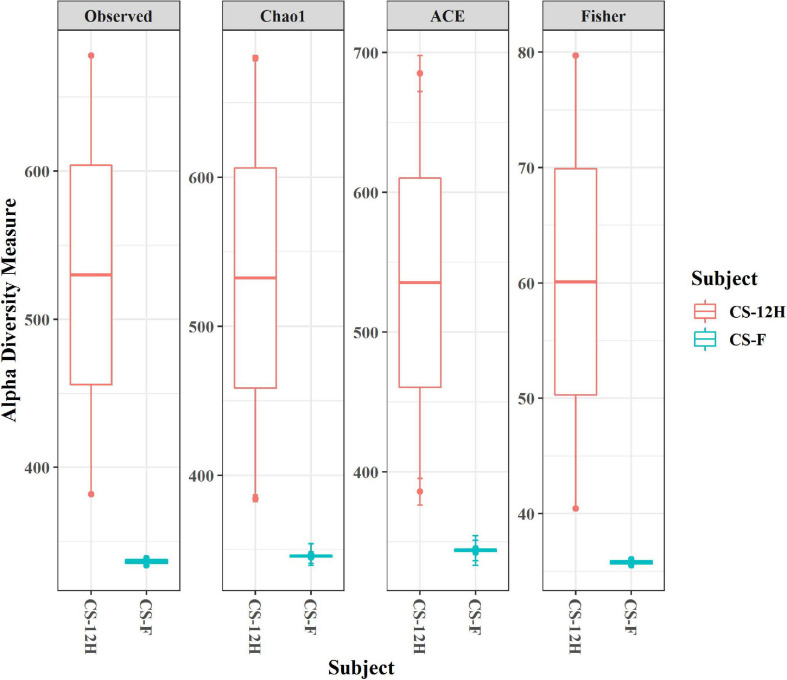
Alpha diversity indices (for observed, Chao1, ACE, and Fisher measures) for fungal reads in fresh (CS-F) and 12-h fermented (CS-12H) *Kalparasa*.

**FIGURE 7:**
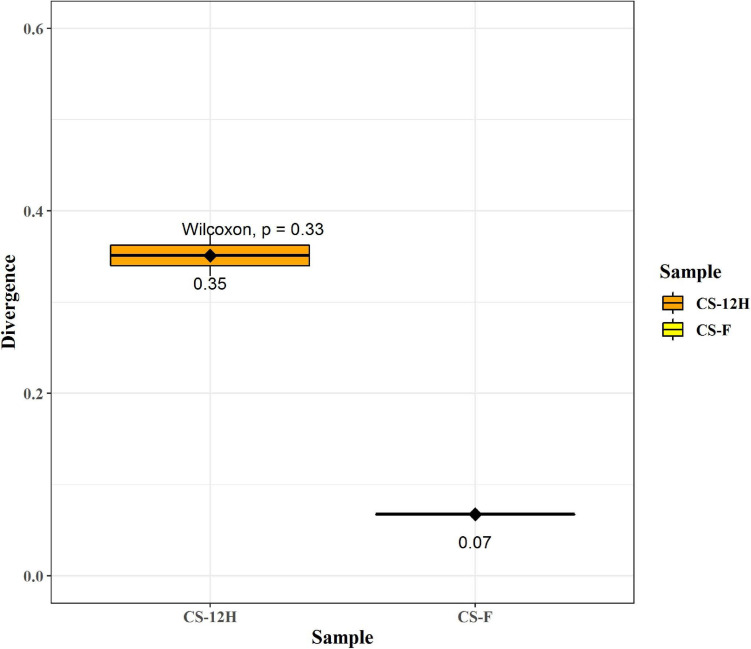
This figure represents group divergence/spread of the given sample set for fungal reads. Here, CS-F (fresh *Kalparasa*) sample divergence is 0.07, whereas CS-12H (12-h fermented *Kalparasa*) sample divergence is 0.33. These can be compared between groups in order to compare differences in group homogeneity.

**FIGURE 8:**
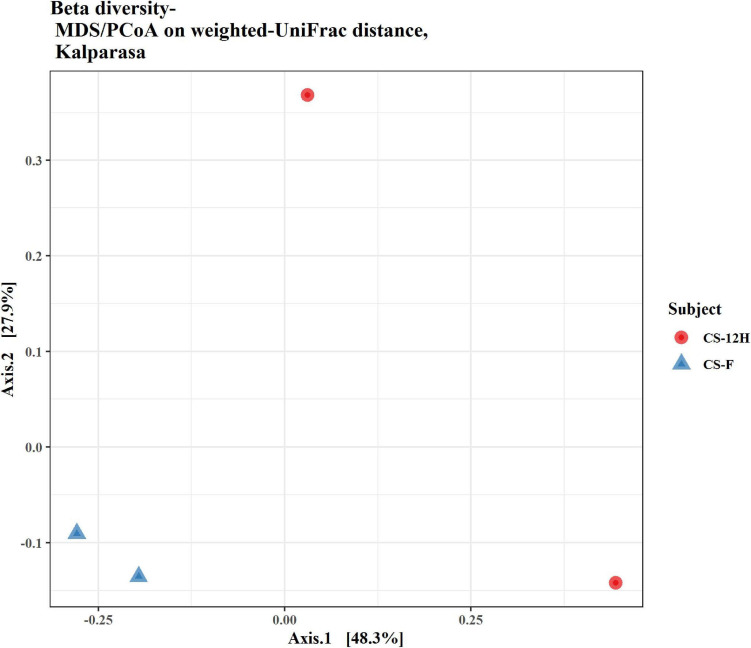
Beta diversity/dissimilarity unifrac distance measure and principal coordinate analysis (PCoA) for fungal reads in fresh (CS-F) and fermented (CS-12H) *Kalparasa*.

The ITS amplification was successful with two samples each of CS-F and CS-12H. We were unable to get amplification for each replication of fresh (CS-F1) and fermented samples (CS-12H3). A total of 1,771,246 chimera-free reads were generated with CS-12H having higher numbers (904,433) than CS-F (866,813) (Table 2). The Venn diagram shows all identified fungal ASVs of CS-F samples and CS-12H, and their interaction, which also indicated additional 251 and 411 fungal ASVs present in CS-F and CS-12H samples, respectively (Supplementary Figure 4). Like bacteria, the *Kalparasa* was dominated by two fungal phyla: Ascomycota and Basidiomycota. However, the fungal classes (9), orders (11), families (25), genera (31), and species (40) were much higher than bacteria (Supplementary Table 4). The most predominant fungal genera in *Kalparasa* were *Saccharomyces, Hanseniaspora, Lachancea*, and *Cortinarius.* CS-F had higher abundance of *Saccharomyces* (54.21%) and *Lachancea* (7.1%) and less of *Hanseniaspora* (28.4%), *Cortinarius* (7.55%), and *Candida* (1.22%). CS-12H had double the abundance of *Hanseniaspora* (42.63%), higher *Cortinarius* (22.1%) and *Candida* (17.82), and very low abundance of *Saccharomyces* (12.04) and *Lachancea* (2.59%) (Supplementary Table 5). Both fresh and fermented *Kalparasa* were rich in the following fungal species: *Saccharomyces cerevisiae*, *Hanseniaspora guilliermondii*, *Lachancea fermentati*, and *Cortinarius saturatus*. *Candida tropicalis* was also one of the highly prevalent fungal species in fermented *Kalparasa* (Figure 5). Log2-fold change values indicated that Ascomycota and Basidiomycota phyla were found 25.72 and 22.99 times, respectively, more in the CS-12H sample (Supplementary Figure 5). The genus-level log2-fold change values are given in Supplementary Figure 6. Fungal diversity analysis indicated that fermented *Kalparasa* was richer in fungal genera based on seven different indices measured (Supplementary Table 6) with the observed, Chao1, ACE, and Fisher clearly highlighting these observations (Figure 6). The divergence data of the CS-F sample was 0.07, whereas CS-12H had 0.33, indicating higher beta diversity values for the former (Figure 7). We generated a PCoA with unifrac distance, which suggested that significant differences existed between the taxonomic compositions of the CS-F and CS-12H sample set, as the first coordinate discriminated them perfectly. The *x*-axis label informed that this separation explained about 48.3% of the variance in the whole dataset (Figure 8).

**TABLE 2:** Fungal preprocessing reads count reduction information for *Kalparasa* samples using the DADA2 R package.

Sample IDs	No. of input reads (raw)	No. of reads after filtering	No. of reads after merging	No. of reads after removal of chimeras
*CS-12H1	653,374	564,186	521,400	510,573
CS-12H2	611,493	485,341	399,241	393,860
**CS-F2	577,460	486,233	443,631	429,587
CS-F3	622,473	497,445	451,812	437,226

*CS-12H (*Kalparasa* sample kept in ambient conditions for 12 h). **CS-F (fresh *Kalparasa*).

### Functional Analysis of *Kalparasa* Bacteriome

**FIGURE 9:**
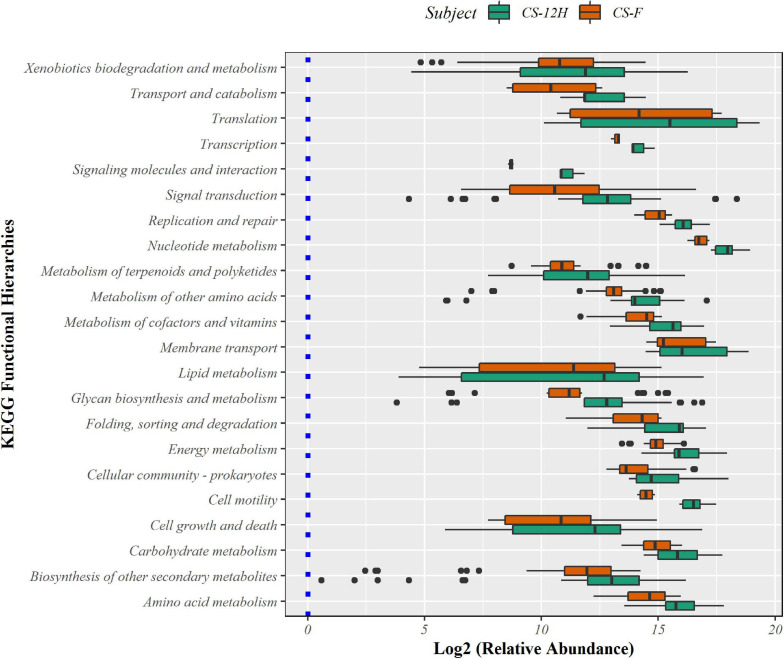
Bar diagram showing abundance box plots for functional hierarchies of KEGG pathway maps present in fresh *Kalparasa* (CS-F) and 12-h fermented *Kalparasa* (CS-12H) samples.

**FIGURE 10:**
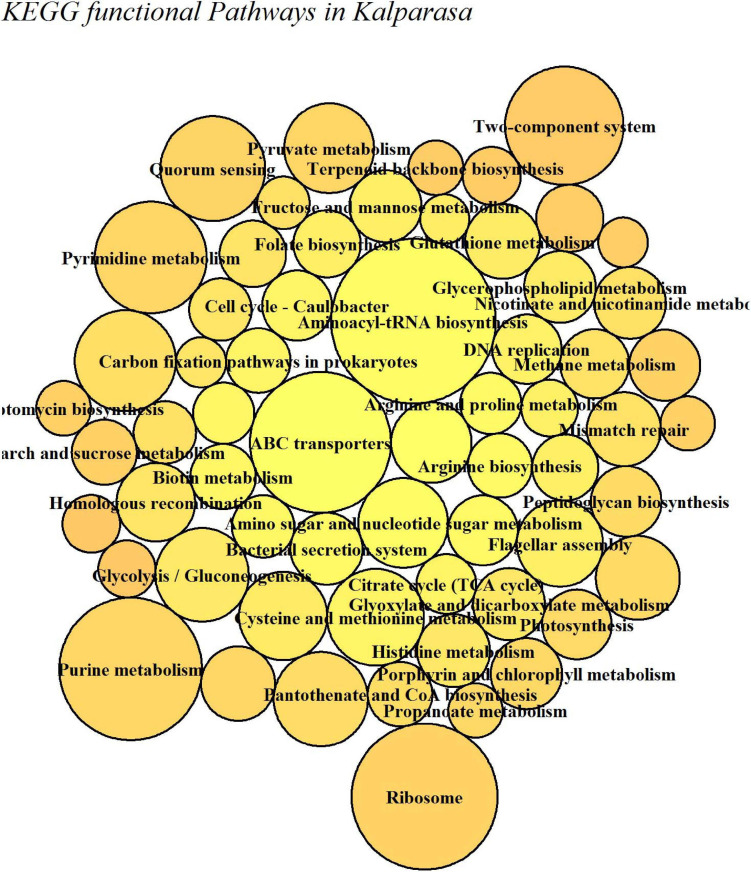
KEGG functional metabolic pathways involved in fresh (CS-F) and 12-h fermented (CS-12H) *Kalparasa*.

The putative functional analysis using KEGG database indicated that *Kalparasa* had a very abundant bacterial activity directed toward the pathways (Supplementary Table 7) responsible for metabolism (68.54% in fresh and 73.5% in fermented samples) and others related to genetic information processing, environmental information processing, and cellular processes in almost equal proportions in both the samples (Supplementary Figure 7). The numbers of functional metabolic enzymes (Supplementary Table 8) involved in the four major pathway classes in *Kalparasa* is furnished in Supplementary Figure 8. Within the pathways, enzymes related to glycerophospholipid, purine, pyrimidine, and pyruvate metabolisms, and ABC transporter as well as two-component systems were seen dominating. Next, ribosome, quorum sensing, and carbon fixation pathways in prokaryotes were prominent (Figure 9). The functional hierarchies based on KEGG pathways reflected the above points with fresh *Kalparasa* having higher functional metabolism on lipid metabolism, translation, cell growth and death, xenobiotic biodegradation, and metabolism of terpenoids and polyketides (Figure 10).

## Discussion

The family Palmae contributes to human nutrition and livelihood in multiple ways. The phloem sap of palms, particularly of palmyra palm (*B. flabellifer*), coconut palm (*C. nucifera*), raffia palm (*Raphia hookeri*), oil palm (*Elaeis guineensis*), and date palm (*Phoenix dactylifera*), is tapped from their unopened inflorescences in several regions in South Asia, Southeast Asia, the Caribbean, South and North America, Africa, and Micronesia for consumption as locally produced alcoholic beverages. The sap is also a rich source of nutrients, minerals, and secondary metabolites, which makes it an edible plant juice. In India, the age-old practice of non-destructive tapping of the inflorescence was adopted to release the phloem sap of coconut and palmyra. The tappers tied an earthen pot, smeared on the inside with lime, to the inflorescence for collecting the sweet sap. The lime helps in slowing down fermentation of the sap. However, despite its application, fermentation begins the moment the sap starts trickling out from the inflorescence into the pot (Hebbar et al., 2013; Endo et al., 2014) due to the action of several microorganisms, particularly the naturally present yeasts, and the fresh sap gets converted to a mild alcoholic drink. In fact, it had been considered that the first stage of fermentation occurred within the receptacle (inflorescence) cut out in the palm tree due to the incision that disturbed the natural microbial population in the biological fermentor (Amoa-Awua et al., 2006). The coconut inflorescence sap collected by traditional methods was reported to be whitish or oyster whitish translucent in color with a sweetish-sour taste (Gupta et al., 1980). The introduction of the coco-sap chiller (patent pending-4077/CHE/2014) by ICAR-CPCRI as an innovative collecting device had shown that the fresh coconut sap is not oyster white but rather golden in color with pH above seven. It had a unique sweet taste with mild coconutty aroma and free of any sour alcoholic flavor and odor (Hebbar et al., 2015). Because of the hitherto unknown uniqueness, the taste and purity of coconut inflorescence sap were brought to light when collected using the coco-sap chiller, termed *Kalparasa*, a product fit for health drink from the *Kalpavriksha* (name used for coconut tree in India as all its parts provide for human needs). The nutritional quality in terms of amino acid, minerals, vitamins, phenols, and secondary metabolites along with the basic microbial profile of *Kalparasa* was studied in detail, which bolstered its distinctiveness and health-promoting properties (Hebbar et al., 2020; Rajesh et al., 2020). However, the complete microbial profile of *Kalparasa* was not assessed thus far. Using the NGS Illumina HiSeq platform, we performed metagenomic analysis to unravel the bacteriome and mycobiome of *Kalparasa*.

**FIGURE 11:**
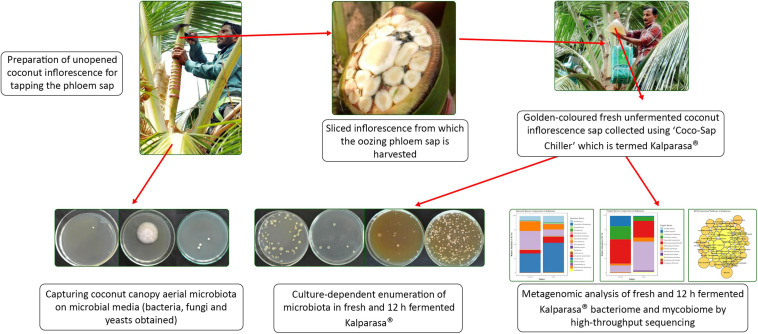
A snapshot graphics capturing the work presented on *Kalparasa* microbiome analysis.

Before the *Kalparasa* was collected for metagenomic studies, we assessed the microbial population circulating in the air around the coconut canopy (Figure 11). A 15-min exposure of the agar plates captured just few microbial colonies, indicating that there was minimum airborne microbiota circulating at the time of *neera* sampling for this study. However, in a previously reported article, several bacteria and yeasts/molds belonging to genera *Acetobacter, Lactobacillus, Saccharomyces*, and *Candida* were isolated from air at different heights of the oil palm (Faparusi, 1973), but the study was conducted with oil palms of less than 8 m height and it was mentioned that closeness of the palm crown to ground and the presence of decaying plant debris in the canopy were the main source of aerial microbiota. In our studies, the coconut palms were more than 15 m tall. The plots were regularly tended to keep it clean of weeds and any litters. Harvesting of the fruits once in 45 days accompanied with canopy cleaning kept the coconut palms and its surroundings in hygienic conditions. This could have been the reason for us not detecting high or diverse microbial population in the selected palms in our studies. Hand sanitization by the tapper and surface sterilization of the inflorescence and coco-sap chiller had minimized entry of microflora from the surfaces that were contacted during the sap harvesting. Our aim to prevent entry of any external microflora from circulating air while fixing the coco-sap chiller or from any other plant parts and capture only the native microflora of the *Kalparasa* was thus ascertained.

Culture-dependent analysis of the fresh and fermented *Kalparasa* clearly indicated the presence of bacteria and yeasts in the samples with the population of yeasts significantly increasing after 12 h of fermentation. The shift in the pH from 7.2 in fresh *Kalparasa* to 4.5 after 12-h exposure to ambient temperature conditions was the direct biochemical indication of fermentation of the sap. Earlier studies of *Kalparasa* kept under refrigerated and ambient conditions also showed a similar change in pH in the latter environment (Hebbar et al., 2018). Thus, the culture-dependent study was able to give a broad idea of the microbial load in the *Kalparasa*. The 16S rRNA amplicon sequencing, on the other hand, could clearly find that fresh *Kalparasa* was dominated by phylum Firmicutes (70.49%), represented more than 60% by the *Leuconostoc* genus, with *L. mesenteroides* being the prominent species. Majority of the ASVs were common to both fresh and fermented *Kalparasa*, while 71 were observed in fresh and 44 were observed in fermented sap. This was indicative of the minimum diversity difference in both the samples and the only difference was the enrichment of species as the fermentation progressed. In a report involving the study of biochemical and microbiological properties of naturally fermenting sap tapped from three different palms (*E. guineensis*, *R. hookeri*, and *Borassus aethiopum*), it was observed that *Fructobacillus* and *Leuconostoc* spp. were preponderant during the 30 days of experiment. The freshly collected sap was acidic in nature with pH ranging between 3.74 and 3.84 (Djeni et al., 2020). In the metagenomic analysis of *tuba*, palm wine produced from coconut inflorescence sap in Mexico, preponderance of Firmicutes and Proteobacteria was reported but with *Fructobacillus* as the dominating genus followed by others such as *Leuconostoc, Gluconacetobacter, Sphingomonas*, and *Vibrio.* The pH of the freshly collected *tuba* was 3.7, which further reduced to 2.8 during fermentation. The freshly collected *tuba*, thus, was already well fermented and enriched in glucose and fructose sugars at the 0-h sample analysis and hence the *Fructobacillus* was seen dominating (Astudillo-Melgar et al., 2019). It is well known that fructose is the preferred sugar for *Fructobacillus* compared to glucose or sucrose and therefore is most abundantly present in fructose-rich niches (Endo et al., 2009). In contrast to *tuba*, fresh *Kalparasa* had an initial pH of 7.2, indicating that the sap was fresh and unfermented and hence had a low abundance of *Fructobacillus.* It was already established that fresh *Kalparasa* had 87% sucrose and a low reducing sugar (0.68 g/100 ml) (Hebbar et al., 2020). *Leuconostoc* prefers sucrose sugar and therefore was found in higher abundance in fresh *Kalparasa*. After keeping fresh *Kalparasa* for 12 h in ambient conditions, the pH fell to 4.5; we noticed that the abundance of phyla Firmicutes (55.65%) and Proteobacteria (44.06%) was closer to each other in the fermented sample. The abundance of the bacterial genus *Gluconobacter* was the highest followed by *Fructobacillus*, whereas *Leuconostoc* was reduced to almost half compared to fresh *Kalparasa*. As the fermentation of Kalparasa proceeded, microbiota dynamics showed partial replacement of *Leuconostoc*, *Acetobacter*, and *Acinetobacter* groups with *Fructobacillus, Gluconobacter*, and *Lactococcus*. In the case of *tuba*, with increase in the fermentation period, the abundance of *Fructobacillus* spp. increased. So, a clear difference in the bacterial profile in *Kalparasa* extracted from coconut palms using the coco sap chiller and that of *tuba* harvested by the traditional method in Mexico was observed. However, our observations of high sucrose content and *Leuconostoc* genus in fresh *Kalparasa* matched with that of *aguamiel*, which is fresh sap collected from agave (Peralta-García et al., 2020). Both *Leuconostoc* and *Fructobacillus* are food-related, obligate heterofermentative LAB together metabolizing glucose, sucrose, and fructose to ethanol, lactate, and acetate. The *Leuconostoc* genus is also widely reported to be present in several nutrient-rich plant parts including flowers and wild as well as fresh fruits and vegetables (Ruiz Rodríguez et al., 2019; Linares-Morales et al., 2020; Ngea et al., 2021). Not only on surfaces of plant parts were *L. mesenteroides, Gluconobacter oxydans*, and few other bacteria present, but also as an endophytic community in healthy leaves of *Agave tequilana*, whose juices are commonly used for the production of tequila (Martínez-Rodríguez et al., 2015). In palms, immature spadix, florets, palm tissues, and leaf surfaces are known to harbor wide varieties of microorganisms including *Lactobacillus* and *L. mesenteroides* (Faparusi, 1971) that could quickly aggregate and colonize in the tissues exposed due to the incision made during the extraction of the nutrient-rich phloem sap from unopened inflorescence. The coconut fruit itself, which is an extension of the developmental stage of the pollinated flower, has been found to possess an endophytic bacterium, *Staphylococcus cohnii*, responsible for the production of beneficial metabolite (Sriram et al., 2020). It is, however, not yet known if LAB are present as endophytes in palm tissues, particularly in inflorescences and leaves. Bacteria such as *Leuconostoc* spp., found in *Kalparasa*, could possess health benefit properties because an earlier report of several LAB isolated from freshly collected and naturally fermenting coconut palm nectar (*neera*) had shown the presence of seven different bacterial species including *Lactobacillus*, *Leuconostoc*, and *Enterococcus* spp. displaying very good probiotic characteristics such as antibiotic traits and hydroxyl radical-scavenging attributes, among others (Somashekaraiah et al., 2019). They were also found to be safe and non-pathogenic to humans based on the *in vitro* hemolytic and DNase activities. *L. mesenteroides* had been reported as one of the main probiotic bacteria in several fresh or fermented food sources such as green peas with an ability to strengthen mucosal barrier through its IgA-inducing ability (Matsuzaki et al., 2013). Kimchi, one of the popular health-promoting Korean food made by fermenting cabbage with other ingredients, was reported to be dominated by the probiotic *L. mesenteroides* bacteria as revealed by metagenomic studies (Jung et al., 2011). The source of *L. mesenteroides* was found to be the garlic added to the cabbage along with other spices during the fermentation (Lee et al., 2015). In addition to *L. mesenteroides*, *Kalparasa* had *Fructobacillus fructosus* and *G. oxydans* in good abundance. *F. fructosus* (*F. fructosus* MCC 3996), like *Leuconostoc*, had also been isolated from the nectar of *Butea monosperma* flower already reported for its probiotic potential (Patil et al., 2020). Fructophilic LAB from such fruits and flower sources were found to possess probiotic potential in ameliorating fructose-mediated irritable bowel syndrome (Sakander et al., 2019). These publications amply support the fact that probiotic LAB are also harbored in fresh unfermented plant products, which may be the case with *Kalparasa* as well.

The mycobiome analysis revealed that *Kalparasa* had more diverse constituents compared to the bacteriome. The mycobiome was represented by 31 genera and 40 species of fungi, whereas the bacteriome was made up of 16 unique genera and 18 species. *Saccharomyces, Hanseniaspora*, and *Lachancea* were the important yeast genera present in the *Kalparasa* at both stages of analysis, albeit with differences in their population. The dominant species were *S. cerevisiae, H. guilliermondii, Lachancea fermentati, C. saturatus*, and *C. tropicalis.* Fresh *Kalparasa* had higher abundance of *Saccharomyces* and *Lachancea* compared with the fermented sample, which had double the abundance of *Hanseniaspora, Candida*, and *Cortinarius* and three-fourths less of *Saccharomyces* compared with the fresh sample. There appeared to be a high number of unique ASVs in the fermented sap than the fresh *Kalparasa* as indicated by the Venn diagram. Population of yeasts was always found in higher abundance in fermented foods, even in coconut sap (Atputharajah et al., 1986) and indigenous yeasts such as *S. cerevisiae* had been widely isolated and used for fermentation research (Udomsaksakul et al., 2018; Limtong et al., 2020). However, it is not uncommon to find large populations of yeasts and bacteria in fresh non-fermented samples too. In studies related to apple cider fermentation (Cousin et al., 2017), yeasts were reported to be present in large numbers in early stages of flower blossom and other plant parts and even up to 4 × 10^8^ cells ml^–1^ in the nectars (Herrera et al., 2009; Pozo et al., 2011). In apple blossoms, the stigma and hypanthium possess a large population of yeasts, which are sometimes even more than bacteria if not similar (Pusey et al., 2009). The above research evidence lends credence to our observations of the presence of high abundance of *S. cerevisiae* in fresh unfermented *Kalparasa*. It can be safely considered that the yeasts such as *S. cerevisiae, H. guilliermondii*, and *Lachancea* present in *Kalparasa* could be having beneficial properties as these yeasts have already been reported as probiotic microbes (Lara-Hidalgo et al., 2017).

The alpha diversity of bacteria and fungi in the *Kalparasa* followed an opposite trend in fresh and fermented samples. The bacterial diversity was seen higher in fresh sap, whereas it had lower fungal diversity when compared to the fermented sample. This clearly showed that the fungi, particularly yeasts, were involved in the metabolism of the sugars at a greater proportion than the bacterial cells. The dominant yeast species detected were *S. cerevisiae, H. guilliermondii, L. fermentati*, and *C. saturatus*. Fresh *Kalparasa* has a clear coconutty aroma and flavor that could be attributed to the presence of *H. guilliermondii* known for producing volatiles that are fruity aromatic in nature (Pietrowski et al., 2012). The fermented *Kalparasa*, conversely, had a strong sour flavor and alcoholic aroma with only a feeble undertone of coconutty odor. GC-MS analysis of fresh, clarified, and fermented coconut sap had shown that the volatiles associated with the typical flavor component of *neera* such as ethyl lactate, phenyl ethyl alcohol, 1-hexanol, 2-methyl tetrahydrofuran, 3-hydroxy-2-pentanone, and 2-hydroxy-3-pentanone increased in concentrations with fermentation. In addition to volatiles, increased amounts of acids such as dodecanoic acid and palmitoleic acid along with higher concentrations of ethyl alcohol and ethyl esters could also abet the harsh and astringent note in the fermented *neera* (Borse et al., 2007). This could perhaps be attributed to the doubling in abundance of *Hanseniaspora* spp. and a concomitant large quantum of volatiles produced by them in the fermented *Kalparasa*. It is probable that the fermentation of *Kalparasa* is driven, along with *Saccharomyces* yeasts, by non-*Saccharomyces* yeasts like *Hanseniaspora* and *Candida*, whose abundance increased significantly after 12 h of keeping the fresh *Kalparasa* in ambient conditions. There are several reports of non-*Saccharomyces* yeast-driven fermentation particularly during the early stages of grape wine with *Hanseniaspora* and *Candida* as the main genera (Zott et al., 2008). In reports on yeasts associated with spontaneous fermentation of *taberna*, a traditional wine prepared from coyol palms (*Acrocomia aculeate*) in Mexico, it was observed that *H. guilliermondii* and *C. tropicalis* were the main yeasts in all the three palm trees sampled whereas *S. cerevisiae* was found only in two of them (Santiago-Urbina et al., 2015). In fermented coconut palm sap, *tuba*, several reports suggest involvement of non-*Saccharomyces* yeasts (Flores-Gallegos et al., 2019). Furthermore, along with the non-*Saccharomyces* yeasts, the bacterial genera such as *Gluconobacter* and *Fructobacillus* could also be involved in the fermentation of the *Kalparasa* as indicated by the microbiome dynamics. This reflects the fermentation that could be driven by symbiotic culture of bacteria and yeasts (SCOBY) as observed in several beverages (Ayed et al., 2020), particularly Kombucha (Villarreal-Soto et al., 2018).

While analyzing the beta diversity of the microbiome of the *Kalparasa*, we noticed that there was variation between the fresh and fermented *Kalparasa*. The bacteria, though varying, clustered more closely to each other than the fungi. In fact, one of the samples for the fungi was very widely spaced from the other. Even though all the palms were of approximately the same age, the same variety, and fertilized with a similar integrated nutrient management regimen, and fruits were harvested in the same frequency and period, the microbiome variation could be explained possibly due to wide genetic heterogeneity that prevails among the West Coast Tall coconut palm (Sudha et al., 2019).

The functional pathway analysis using the KEGG database indicated that *Kalparasa* had significant bacterial activity directed toward the metabolism (68.54% in fresh and 73.5% in fermented samples) and equal proportions of genetic information processing, environmental information processing, and cellular processes. This was obvious because the *Kalparasa* was rich in sugars, amino acids, and phenols (Hebbar et al., 2020), which the bacteria were actively utilizing for their metabolic activity and multiplication. We observed that the glycerophospholipid metabolism in the *Kalparasa* was strongly represented perhaps due to the presence of lipid contents to the tune of 12 as evidenced by MS analysis of the sap (Rajesh et al., 2020). Another key metabolic pathway related to *Kalparasa* was the two-component system. This pathway is ubiquitously present in bacteria and is used in cell-to-cell signaling for chemotaxis, osmotic sensing, and light perception changes in environment (Stock et al., 2000). It is obvious that there will be significant shifts in environmental attributes when coconut inflorescence sap emerges from plant cells, then gets collected in the coco-sap chiller and further left in ambient conditions for 12 h. These changes in the environmental conditions might be activating the two-component system metabolism in the bacteriome in *Kalparasa* and propel the microbes to cause fermentation and other associated changes in the fluid. Quorum sensing (QS) was also seen as a dominant metabolic activity in both the samples of the *Kalparasa*. Yeasts have been reported to be involved in QS in fermented food for possibly regulating their community structure (Johansen and Jespersen, 2017). Among yeasts, *S. cerevisiae* is known to switch on its QS activity in response to external stimuli in the form of changing environmental conditions such as changing cell density, ethanol, nitrogen content, and oxygen status (Avbelj et al., 2016), which *Kalparasa* collection also encounters.

### Source of *Kalparasa* Microbiome?

Thus, metagenomic analysis conducted by us with limited palm samples indicated the presence of abundant and diverse microbiome in fresh unfermented *Kalparasa*. However, the source of this microbiome is unclear, and it can be hypothesized that the microbiome in fresh *Kalparasa* could have arrived from endophytic or epiphtyes in floral portion of the inflorescence, or from the atmospheric air or a combination of all these sources. This needs to be further researched. Moreover, because of an enclosed collection apparatus, the transfer of cold temperature from the coco sap chiller to the spadix connecter and the spadix must play an important role in preventing the disturbance of natural microflora and thus avoid the initiation of souring within the biological fermenter (inflorescence) normally associated with the traditional method of sap collection. A better understanding of the microbiome can be obtained by analyzing Kalparasa from more number of coconut palms along with the associated plant parts.

### Is *Kalparasa* a Product of Immortal Plant Cells?

Longevity of any cell or its lifespan is determined by the period over which the constituent cells remain metabolically functional. Palms, such as coconut, have primary growth living cells that remain metabolically active throughout its lifetime. In a broad sense, palm cells, particularly the sieve tubes and their companion cells, are therefore deemed immortal (Tomlinson and Huggett, 2012). Though the authors do not claim for any chemical reasons for the cell longevity and only highlight the distinct developmental features of palm cell but with current information of the health-promoting biochemical and microbial constituents in *Kalparasa*, it could be worthwhile to examine its effect on cell longevity.

## Conclusion

The 16S rRNA and ITS amplicon sequencing-based metagenomic analysis of *Kalparasa* disclosed the elaborate diversity of bacteria and yeasts harbored in the coconut inflorescence sap collected using the novel “coco-sap chiller.” Fresh *Kalparasa* had a pH of 7.2, indicating the non-fermented status, accompanied with high abundance of lactic acid bacteria (LAB), *Leuconostoc* spp., and *Fructobacillus* spp., and yeasts such as *Saccharomyces* spp., *Hanseniaspora* spp., and *Lachancea* spp. The dominant species were *L. mesenteroides, F. fructosus, S. cerevisiae*, and *H. guilliermondii* in fresh *Kalparasa.* Keeping the *Kalparasa* in ambient conditions for 12 h resulted in lowering of pH to 4.7 accompanied with sour alcoholic aroma indicating fermentation. A shift from LAB to acetic acid bacteria (AAB) with *Gluconobacter* and *Fructobacillus* spp. dominating the bacteriome density while *Hanseniaspora* spp. was highest in mycobiome demonstrated that AAB and non-*Saccharomyces* yeast could be driving the fermentation of *Kalparasa*. This reflected that the fermentation could be driven by SCOBY. Our studies clearly indicated that like fermented palm saps harbored probiotic microbiome, fresh *Kalparasa* also had abundant probiotic-type microbiome consisting of *L. mesenteroides, F. fructosus, S. cerevisiae*, and *H. guilliermondii.* These findings add value to promote *Kalparasa* as a natural health drink full of beneficial properties arising from its probiotic-type microbiome.

## Data Availability Statement

The datasets presented in this study can be found in NCBI BioProject ID: PRJNA701871.

## Author Contributions

MG and AG designed and conducted the study and obtained the data. SS and MG carried out the bioinformatic analysis. SS did the statistical analysis in R-package. KBH, the original developer of coco-sap chiller and *Kalparasa*, and MA were involved in the investigation. MG, SS, and AG wrote the manuscript with inputs from all authors. All the authors contributed to the article and approved the submitted version.

## Conflict of Interest

The authors declare that the research was conducted in the absence of any commercial or financial relationships that could be construed as a potential conflict of interest.

## Publisher’s Note

All claims expressed in this article are solely those of the authors and do not necessarily represent those of their affiliated organizations, or those of the publisher, the editors and the reviewers. Any product that may be evaluated in this article, or claim that may be made by its manufacturer, is not guaranteed or endorsed by the publisher.
