# Supplementary material for: Metagenomic Investigation Uncovers Presence of Probiotic-Type Microbiome in Kalparasa® (Fresh Unfermented Coconut Inflorescence Sap)
Source: Front Microbiol. 2021 Aug 13;12:662783. doi: 10.3389/fmicb.2021.662783 (PMC8415118; doi:10.3389/fmicb.2021.662783)
Supplement: Supplementary file 2 [file Data_Sheet_1.docx]

Metagenomic investigation uncovers presence of probiotic-type microbiome in Kalparasa^®^ (fresh unfermented coconut inflorescence sap)

Murali Gopal^1^*, Sandip Shil^2^, Alka Gupta^1^, K. B. Hebbar^1^, M. Arivalagan^1^ ^‡^

^1^ICAR-Central Plantation Crops Research Institute, Kasaragod, Kerala, 671124, India

^2^ Research Centre, ICAR-Central Plantation Crops Research Institute, Mohitnagar, West Bengal, India

^‡^ Present Address: ICAR-Indian Institute of Horticultural Research, Bengaluru, Karnataka, India

**Supplementary Figures**


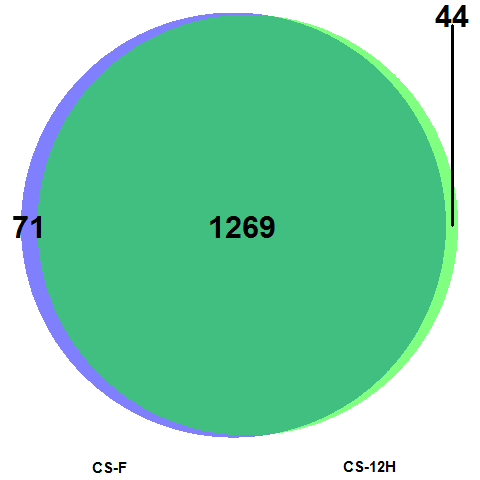

**Suppl. Fig S1.** Venn diagram showing bacterial status of fresh *Kalparasa* (CS-F) samples on the left, fermented *Kalparasa* (CS-12H) on the right and their interactions.


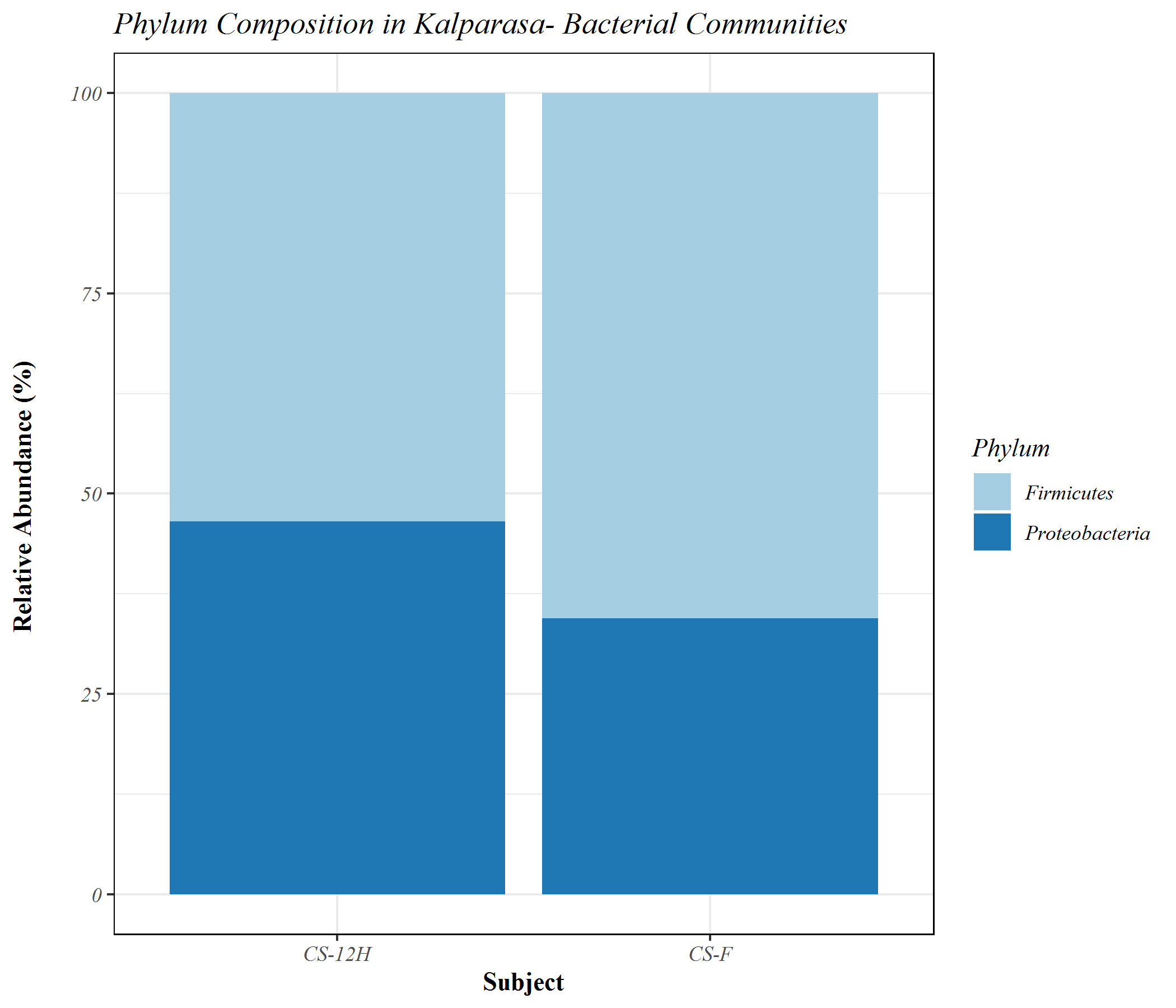


**Suppl. Fig S2.** Bar plot representing phylum wise abundance for bacterial reads in fresh (CS-F) and fermented (CS-12H) *Kalparasa*


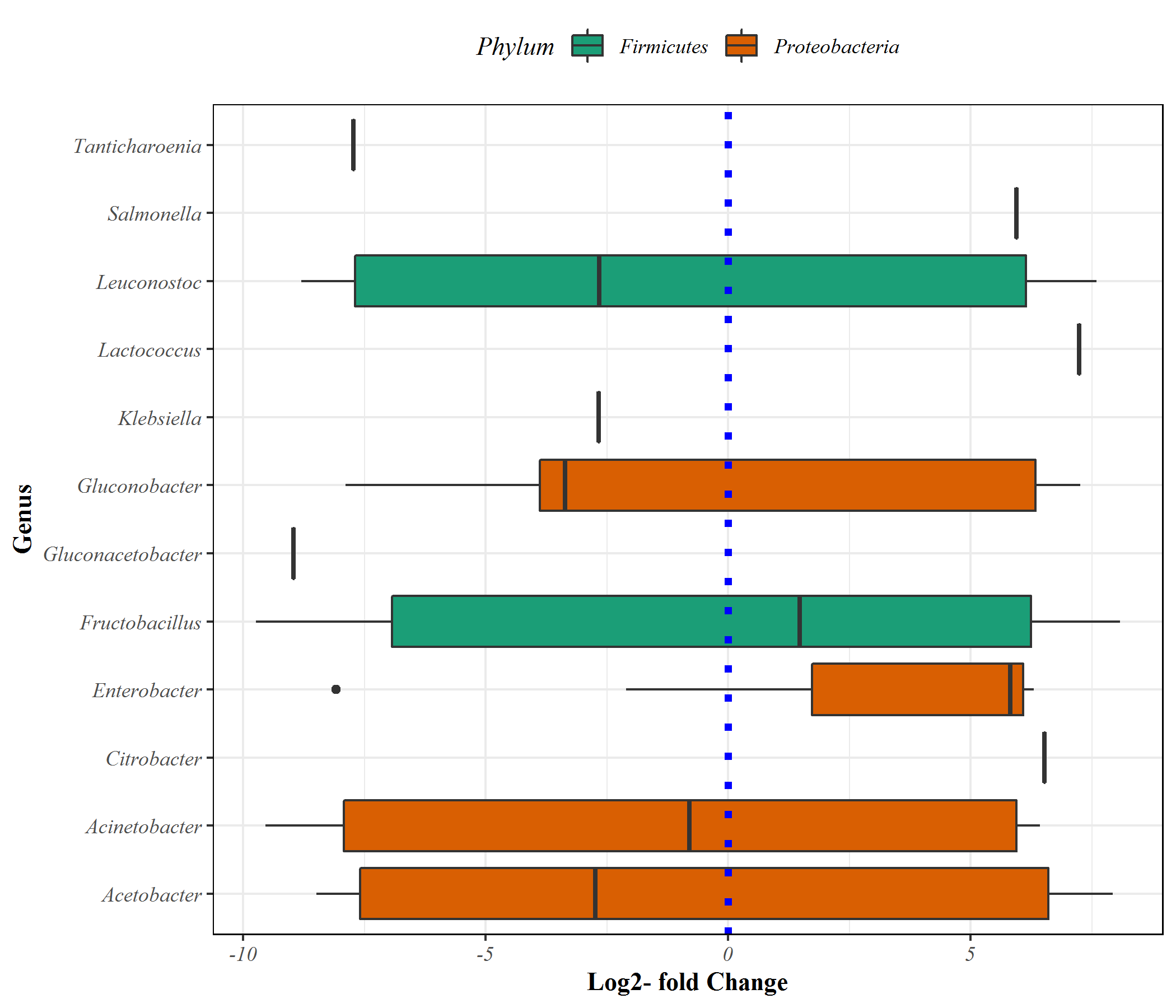


**Suppl. Fig S3.** Bar plot representing genus level- log2 fold abundance in fresh (CS-F) and fermented (CS-12H) *Kalparasa* for bacterial reads


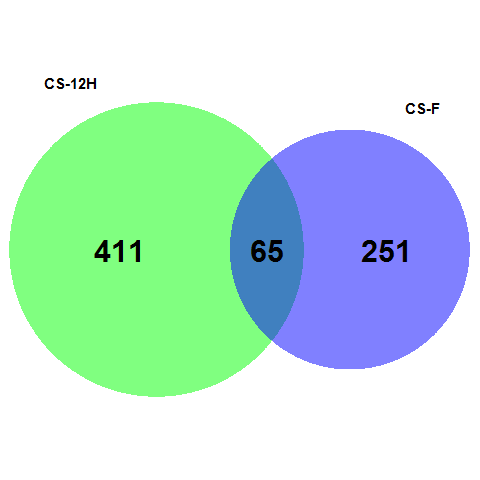


**Suppl. Fig. S4:** Venn diagram showing fungal status of fresh *Kalparasa* (CS-F) samples on the right, fermented *Kalparasa* (CS-12H) on the left and their interactions.


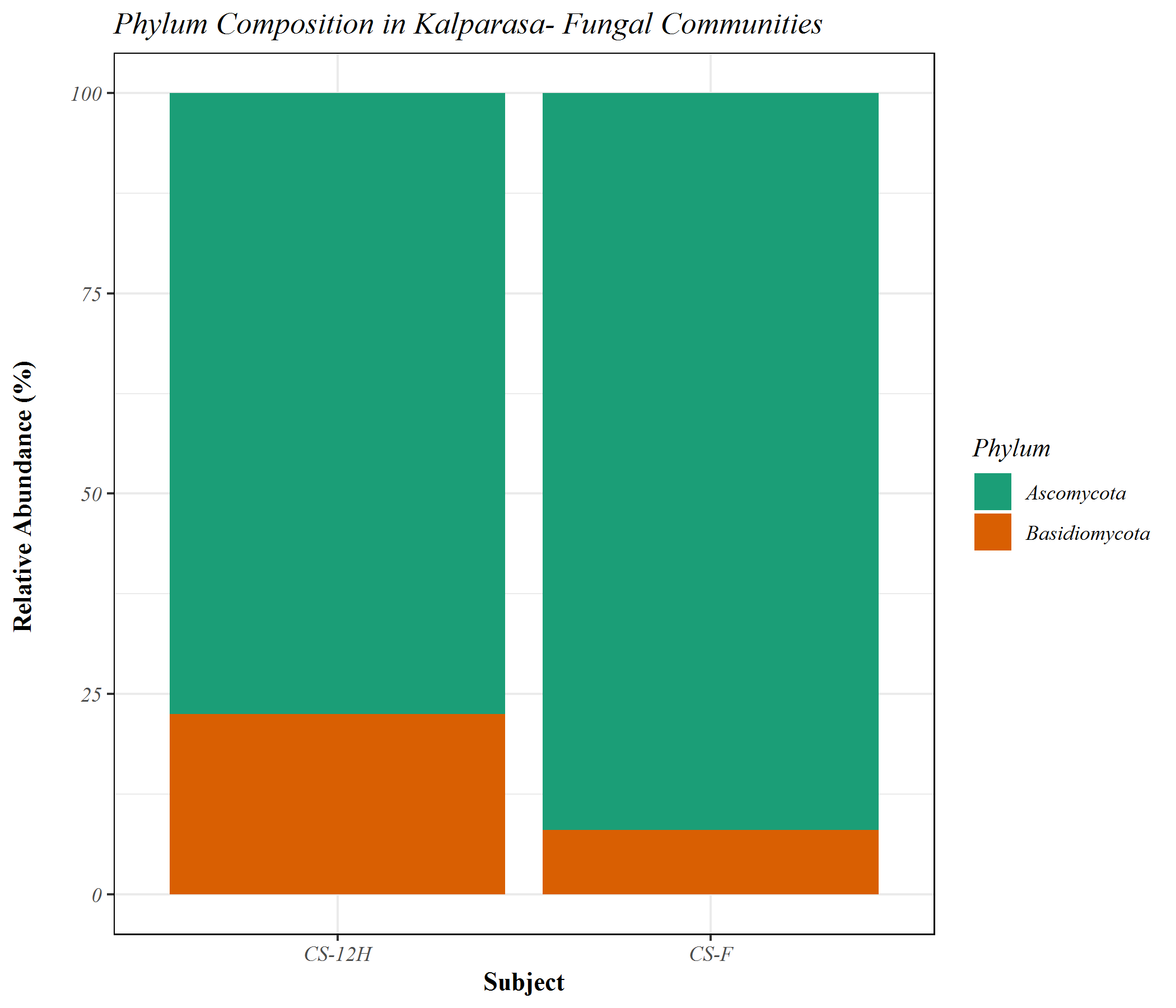


**Suppl. Fig S5.** Bar plot representing phylum wise abundance in fresh (CS-F) and fermented (CS-12H) *Kalparasa* for fungal reads


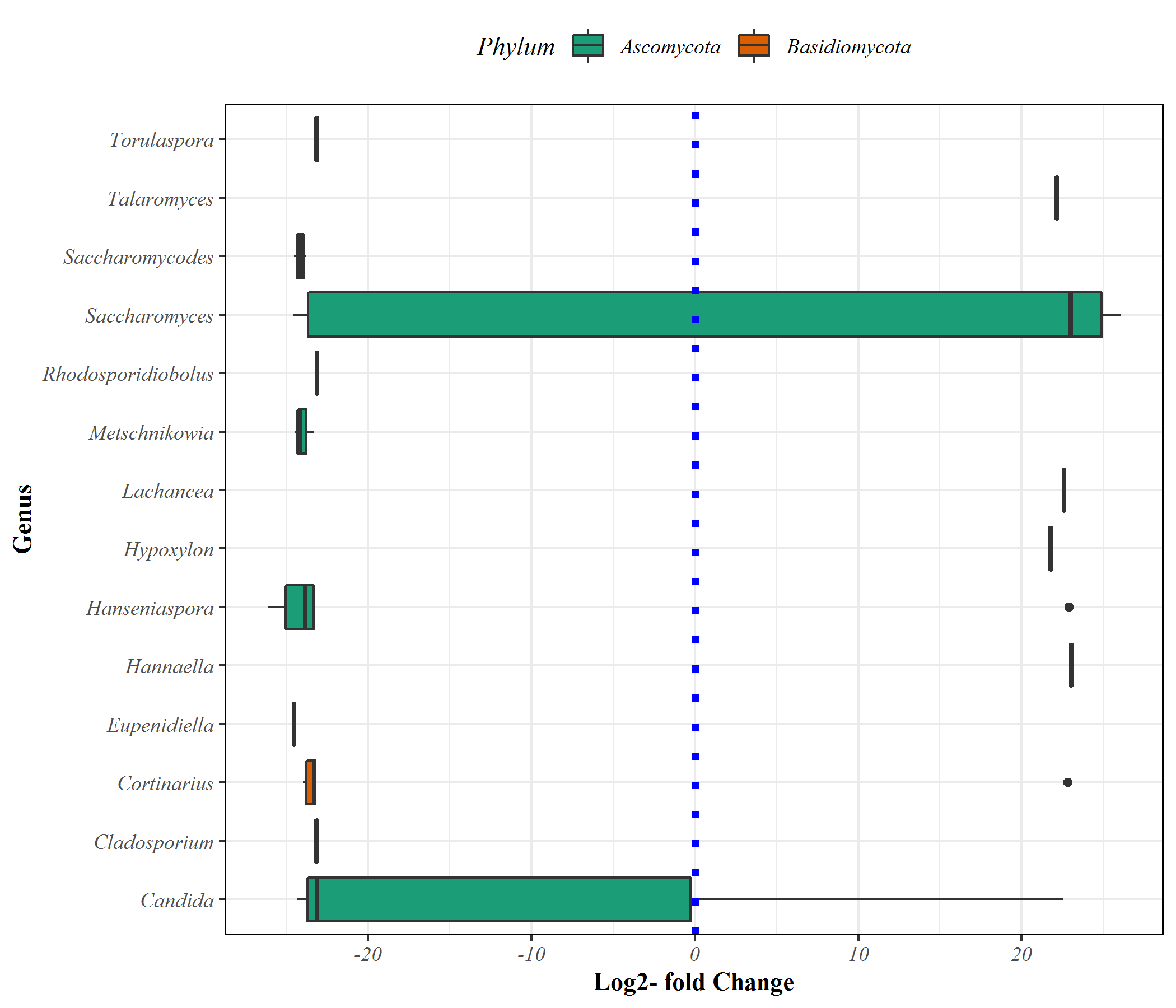


**Suppl. Fig S6:** Bar plot representing genus wise abundance at level- log2 fold changes in fresh (CS-F) and fermented (CS-12H) *Kalparasa* fungal reads


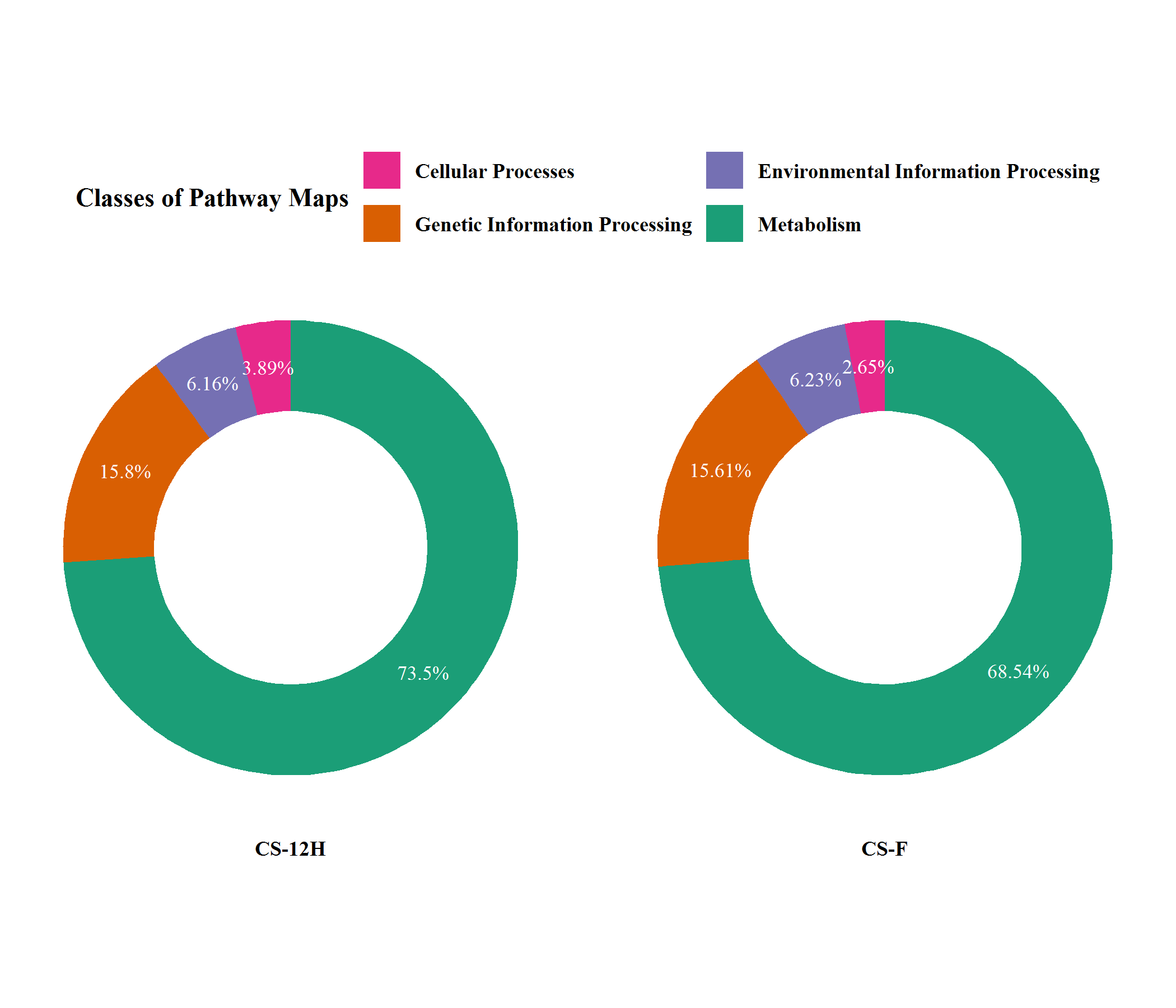


**Suppl. Fig. S7:** Donut diagram showing abundance percentages of classes of KEGG pathway maps in fresh (CS-F) and fermented (CS-12H) *Kalparasa*

**
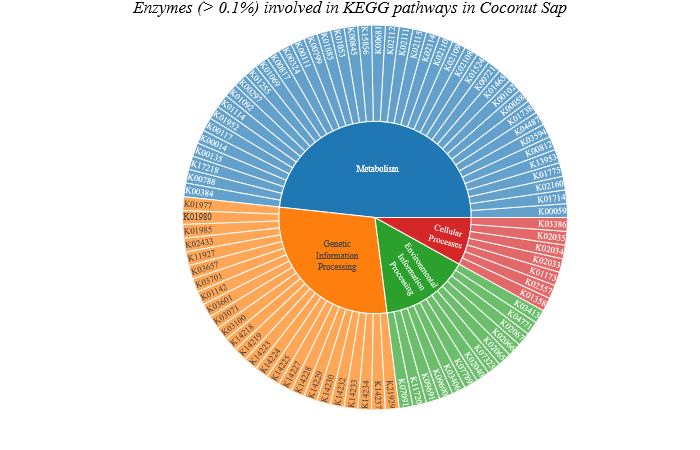
**

**Suppl. Fig. S8:** KEGG functional metabolic enzymes contributing >0.1 % involved in KEGG Pathways in fresh (CS-F) and fermented (CS-12H) *Kalparasa*
